# ‘We should have been told what would happen’: Children’s and parents’ procedural knowledge levels and information-seeking behaviours when coming to hospital for a planned procedure

**DOI:** 10.1177/13674935211000929

**Published:** 2021-03-20

**Authors:** Lucy Bray, Victoria Appleton, Ashley Sharpe

**Affiliations:** 1 Faculty of Health, Social Care and Medicine, 6249 Edge Hill University, Ormskirk, UK

**Keywords:** Child, access to information, information seeking, procedure, hospital, parents

## Abstract

Children continue to be poorly prepared and informed about clinical procedures, despite increased evidence of the worth of preparation and the availability of information resources. This study used a concurrent mixed-methods approach to explore the information accessed by children and their parents before attending hospital for a procedure. Information was collected separately from 40 children (aged between 8 and 12 years) and their parents using a paper booklet to examine self-reported perceived procedural knowledge and information-seeking behaviours. Data were analysed using descriptive statistics and content analysis techniques. The findings indicate that many children (70%, *n* = 28) and their parents (65%, *n* = 26) have low procedural knowledge levels. The majority of children (85%, *n* = 36) reported not receiving or seeking information about their procedure, despite identifying a desire and preference for more information. This study shows a mismatch between the current provision of procedural information and children and parents’ expectations that information will be provided directly to them by health professionals. In order for this ‘information hole’ to be filled, there needs to be a concerted effort to develop and systematically use meaningful information materials and for children and their parents to have the opportunity to discuss their procedural knowledge with health professionals.

## Background

Children are key users of health services, with over six million outpatient attendances for 5–14 year olds in the United Kingdom in 2017–2018 (NHS Digital, 2018) and over 65,000 children undergoing surgery (NHS Digital, 2018). Children can find visiting a hospital for a planned clinical procedure such as a blood test, radiological procedure or surgery, a stressful, disorientating and fearful experience (Duff et al., 2012; Justus et al., 2006; Kain et al., 2006, 2007). Children’s anxiety can arise from fear of the unknown (Clift et al., 2007), concerns over separation from parents (Platt et al., 2016), loss of control (Claar et al., 2002; Koukourikos et al., 2015) and a lack of knowledge and preparation about what will happen (Gordon et al., 2011; Jaaniste et al., 2007).

There is strong evidence to show that children have a better experience (less anxiety, less upset and less likely to be held) if they are well prepared and informed about what will happen when they attend hospital for a procedure (Bray et al., 2015, 2016, 2019b; Copanitsanou and Valkeapää, 2014; Coyne et al., 2016; Oulton et al., 2018), and the importance of preparatory information is acknowledged by national (Royal College of Nursing, 2019) and international bodies (European Association of Children in Hospital). If children are not provided with information, then they can develop inaccurate understandings about what will happen (Bray et al., 2019a, 2019b; Lambert et al., 2008). Children have recognised rights to receive information, to be listened to and to be facilitated to join in decisions of relevance to them (United Nations General Assembly, 1989), yet there is still progress to be made in what we understand about children’s preparation for procedures in hospital.

Much of the research examining the impact of preparation and information programmes has focussed specifically on children undergoing surgery (Fernandes et al., 2014; Kain et al., 2007, 2015; Li and Lopez 2008; Wright et al., 2017) or invasive procedures such as cannulation (Tunç-Tuna and Açikgoz, 2015), using methods such as books (Felder-Puig et al., 2003), online information (Fortier et al., 2015; Williams and Greene, 2015; Wright et al., 2017) or specialist play staff (Li et al., 2007). The provision of information within these structured research studies is often conducted by dedicated staff which can be above and beyond normal everyday clinical practice. There is less known about what information children and their parents access and use outside of these focussed programmes. Despite increased evidence of the worth of preparation and the quantity and quality of resources for providing information, children continue to be poorly prepared and informed about their procedure (Bray et al., 2019b; Smith and Callery, 2005).

There are challenges in how children can access meaningful information. Constraints within clinical services means that there can be little time or opportunity for health professionals to provide information or prepare children for their procedure. Even though we know that children value information delivered directly from health professionals (Buford, 2005; Lambert et al., 2008). Currently, children often only receive information from healthcare providers via their parent/carer (Bray et al., 2016), and this can lead to parents having to shoulder the responsibility for informing and preparing their child (Bray et al., 2019b). This can be problematic if parents are unfamiliar with aspects of procedures, ill-equipped to know what to say to their child (Bray et al. 2019b; Gordon et al., 2011; Smith and Callery, 2005), choose not to engage with information resources (Bray et al., 2019b) or filter information to limit their child’s access to information seen as potentially anxiety provoking (Young et al., 2003). The role of parents in filtering information to their child or acting as an intermediary body (Lambert et al., 2008) or information broker (Young et al., 2003) is seen in many different contexts, including children’s inpatient admission (Lambert et al., 2008), cancer care (Young et al., 2003) or relating to broader health concerns such as COVID-19 (Bray et al., 2021). This paper explores children and their parent’s access to information and their information preferences prior to attending hospital for a planned procedure.

## Aim

To understand more about the procedural information accessed by children and their parents before attending hospital for a planned procedure.

## Research design

### Participants

Children and their parents were recruited from one regional children’s hospital in England. A purposive sampling technique (Etikan et al., 2016) was used to gain maximum variation in children’s age, gender and planned procedure. Eligible families were invited to take part in the study by their clinical team; eligible children were those who were due to attend hospital for a planned procedure in the next few weeks, were aged 8 to 14 years, were not under the care of psychological services for procedural anxiety and had no moderate or severe cognitive impairment. Children and their parents were provided with information leaflets about the study and allowed time to ask questions and consider participation. If a child and parent agreed to take part, the researcher met them when they attended hospital for the planned procedure and obtained parental consent and assent from the child.

## Research methods

This research study used a concurrent or convergent mixed-methods approach (Creswell et al., 2007) where quantitative questionnaire data and qualitative interview data were collected in parallel. The qualitative data aimed to help provide some explanatory understanding of the quantitative data and as such was an embedded element to the study design (Creswell et al., 2003). The data presented were collected from a group of children who received standard hospital information provision as part of a larger cohort study which was focussed on the evaluation of a gamification and augmented reality health app (Xploro, 2020; https://xploro.health/) for children attending hospital (Bray et al., 2020). Fieldwork was carried out over a 4-month period in 2018.

### Self-report questionnaire

A paper questionnaire was used to measure children and parents’ self-reported knowledge levels and access to procedural information. The questionnaire consisted of structured closed self-report questions (see Table 1 for further detail). Children and their parents were asked on a visual analogue scale (0–10) to rate how much knowledge they perceived they had about the planned procedure and asked to agree or disagree with a statement about their knowledge levels. Children were additionally asked to self-report what information sources they had accessed prior to attending hospital and their information preferences based on some elements from the Procedural Coping Questionnaire (PCQ; Phillips et al. 1998). Questionnaires were administered as children and their parents waited for their procedure within the clinical department.

**Table 1.** Structured questions on the questionnaire.

	Topics	Questions on the children’s questionnaire	Questions on the parents’ questionnaire
*Children and their parents’ procedural knowledge levels*	Perceived procedural knowledge level	*How much do you know about the procedure you are having today and what is going to happen?*	*How much do you know about the procedure your child is having today and what is going to happen?*
		Visual analogue scale 0–10 (0=know nothing, 10=know everything)	Visual analogue scale 0–10 (0=know nothing, 10 =know everything)
	Perceived procedural knowledge level	*Do you know enough about what is going to happen to you today?*	*I know enough about the procedure my child is having today*
		Yes/no/don’t know	Agree/disagree/don’t know
	Perceived information level	*I have had enough information about what is going to happen to me today*	*I have had enough information about the procedure my child is having today*
		Agree/disagree	Agree/disagree
*Children and parents’ information preferences and access to information*	Access and use of information before attending hospital for the procedure	*Did you look at any sources of preparation/information before you came to hospital?*	Not asked
		*Which sources did you look at?*	
		Website/app/leaflet/talking to friends or family/book/others	
		*How often did you access of look at this source?*	
		Number of times and length of time	
		*Was this source of information useful?*	
		Yes/no	
	Information preferences and information-seeking questions based on some elements from the Procedural Coping Questionnaire (PCQ) (Phillips et al., 1998)	*I like to know as much about it as possible*	Not asked
		Yes/no/don’t know	
		*I talk to the doctor and nurse before they start so I know exactly what is going to happen*	
		Yes/no/don’t know	
		*I would rather not know what is going to happen*	
		Yes/no/don’t know	
		*What did you do today which helped you get through your procedure?*	
		Free text	

### Qualitative interview

A short semi-structured qualitative interview sought to explore what children and their parents thought had worked well during the procedure and what could have made it better. The interview used activity sheets with thought and speech bubbles. As advocated by Noonan et al. (2016), children and parents could choose whether to write their responses on the activity sheet or tell the researcher their opinions and thoughts. The interview was conducted separately with children and their parents within a quiet area in the clinical department after the child’s procedure had been completed.

### Patient and public involvement and engagement

We consulted with children, young people and parents during the development of the research study. This is reported according to GRIPP2 short form (Staniszewska et al., 2017). This patient and public involvement and engagement (PPIE) aimed to inform the design of the study and ensure our recruitment approaches, study information and the content and format of data collection methods were appropriate and accessible for children and their parents. We conducted two face-to-face workshops with 10 children and young people aged 12 to 13 years with varied experience of health services and individual consultation with four parents. Our PPIE informed the design and content of the questionnaire booklet, the inclusion and presentation of the measurement scales (Likert and VAS), the questions and format used on the activity sheets and the language and layout of the study information to enable them to be more engaging and understandable for children and parents.

## Analysis

In line with the convergent mixed-methods design, the quantitative and qualitative data were analysed separately and then merged or integrated (Fetters et al., 2013) under the main foci of procedural knowledge and information preferences. The quantitative data were analysed using descriptive statistics (frequencies and percentages) using SPSS (v25, 2017). Qualitative responses were analysed by content analysis techniques (Hseih and Shannon, 2005), the research team members independently coded the data inductively and the developed codes were then discussed and themes were created through consensus making processes.

## Ethics approval

This study was awarded ethical approval by the researcher’s institution (REF code and institution to be added after peer review) and the Health Research Authority (18/WA/0277). Written consent was obtained from all parents, and written or verbal assent was obtained from all children. All participant responses were anonymised on the write and tell sheets.

## Results

### Demographics

Forty children and their parents who received standard hospital information were recruited to the study. The children were aged 8–14 years (mean age 11, SD 1.8), 19 were female and 21 were male. The children were attending hospital for a range of procedures including surgery (30%, *n* = 12), X-ray (17.5%, *n* = 7), cast removal (7.5%, *n* = 3), stitch removal (27.5%, *n* = 11), stitch removal (10%, *n* = 4), MRI scan (7.5%, *n* = 3) and an ultrasound scan (2.5%, *n* = 1). For the majority of the children (83%, *n* = 33), it was the first time they were coming to hospital to have the particular procedure conducted, although the children had a range of previous hospital experiences, with many (55%, *n* = 22) reporting minimal experience of hospitals (between 0 and 3 previous hospital attendances). All the children and parents who were invited to take part completed data collection.

## Findings

Our findings are presented according to a ‘weaving approach’ (Fetters et al., 2013) where both qualitative and quantitative findings are presented together within the two key themes which underpinned the foci of the study: ‘I didn’t know what was going to happen’: levels of procedural knowledge, and ‘We should have been told and didn’t have time to ask’: information access and preferences.

### ‘We didn’t know what was going to happen’: levels of procedural knowledge

The majority of children (70%, *n* = 28) and their parents (65%, *n* = 26) reported that they did not have enough information about the planned procedure (Table 2) and reported poor levels of perceived procedural knowledge. As described in the methods section, children and their parents were asked to rate how much knowledge they perceived they had about the planned procedure on a VAS (0–10). Most children (75%, *n* = 30) and their parents (60%, *n* = 24) reported having low to medium levels of knowledge about the planned procedure (Table 3). Children’s median perceived knowledge was 3.5 (interquartile range (IQR) 3), and parents’ median was higher at 5.5 (IQR 3.25).

**Table 2.** Perceptions of levels of information and knowledge.

Perceptions of information levels		Agree *n* (%)	Disagree *n* (%)
Child	I have had enough information about what is going to happen to me today	12 (30)	28 (70)
Parent	I have had enough information about the procedure my child is having today	14 (35)	26 (65)
*Perceptions of knowledge levels*		*Agree*	*Disagree*
Child	I know enough about what is going to happen to me today	12 (30)	28 (70)
Parent	I know enough about the procedure my child is having today	16 (40)	24 (60)

**Table 3.** Children and their parents’ reported perceived knowledge levels.

	Low knowledge levels (0–3 on the VAS), *n* (%)	Medium knowledge levels (4–7 on the VAS), *n* (%)	High knowledge levels (8–10 on the VAS), *n* (%)
Children	18 (45)	12 (30)	10 (25)
Parents	6 (15)	18 (45)	16 (40)

Many of the children and parents shared in the interviews that they did not know what was happening during their hospital visit:

“*The nurse didn’t say anything she just did things and I didn’t know what any of it meant*” (P24, child).

Many of the responses to the ‘what could have been better’ question related to information and preparation, with both children and their parents identifying that they would have liked to have known more information:

“*They could have prepared us a bit more by explaining before we came in what would happen*” (P20, parent).

This lack of knowledge and preparation was described as impacting negatively on children and their parents:

“*Mum didn’t know what would happen so she got upset*” (P29, child).

### “We should have been told and didn’t have time to ask”: accessing procedural information

Children were asked what information they had received, looked at or accessed before coming to hospital for their procedure. This could be reading the information sent from the hospital, accessing either online or written information or talking about the procedure with family or friends (Table 4). Nearly all children (85%, *n* = 36) reported not receiving, accessing or seeking information about their procedure. Out of the 12 children attending for surgery, only two reported having read the leaflet they had been given; even though all families at the participating hospital handed out information leaflets at the preoperative assessment clinic. One of the children who had accessed information online had found it really useful:

“*I should have shown my mum the YouTube video as well then she would have known what to expect*” (P21, child).

**Table 4.** The information children looked at before attending hospital for procedures.

Information source	Number of children who accessed information sources prior to attending hospital, *n* (%)
Website	3 (8)
App	0 (0)
Leaflet	2 (5)
Family/friend	1 (3)
Book	0 (0)
Others	0

The low number of children accessing information was at odds with their responses to questions regarding their information-seeking behaviours drawn from the PCQ (Phillips et al., 1998). Nearly all of the children (92.5%, *n* = 37) reported a preference to know what would happen during a procedure and stated that they would actively try and gain information from health professionals before their procedure started (97.5%, *n* = 39) (Table 5).

**Table 5.** Information-seeking questions and children’s responses.

	Number of children who agreed with this statement, *n* (%)
I like to know as much about a procedure as possible	37 (93)
I would rather not know what is going to happen	1 (3)
I talk to the doctor and nurse before they start so I know exactly what is going to happen	39 (98)

The interviews highlighted that in many cases children and their parents had expected information to be provided by health professionals. Children and their parents identified wanting more information about what was due to happen during their hospital visit, as identified by the following parent:

“*We could have had some information on what an ultrasound is like for a child*” (P26, parent) Children also discussed wanting more information about what would happen, demonstrated by the following two participants:

“*I could have been told I would have two appointments*” (P28, child),

“*No-one told me much*” (P6, child).

This lack of procedural information meant that children and their parents were not able to prepare themselves for what would happen at the hospital, as expressed by the following two parents:

“*We could have been told stitches were coming out today*” (P12, parent),

“*We should have been told about numbing cream on last appointment*” (P21, parent).

In all the instances, children and parents identified health professionals as the main expected source for information. However, many of the children and parents acknowledged the difficulty of gaining information at the point of admission; one child commented that they

“*Had felt rushed and I didn’t feel like I could ask anything*” (P23, child).

Parents also commented on feeling there was a lack of time on admission, highlighted by the following two parents:

“*We felt rushed being shown round the ward and explained things to*” (P24, parent)

“*We should have had more time and felt less rushed”*(P2, parent).

The children particularly seemed to value instances where health professionals had found time to share information with them as demonstrated by the two children below:

“*The lady was nice and showed me my knee and told me what everything was*” (P26, child),

“*The nurse was kind and explained it was like a straw and not a needle*” (P10, child),

Time spent preparing and explaining a procedure to a child was also positively discussed by parents, as highlighted by the following parent:

“*The X-ray lady was lovely and took time to explain things to him*” (P8, parent).

## Discussion

This study aimed to examine the procedural information accessed by children and their parents before attending hospital for a planned procedure. Our findings add further understanding to children’s procedural preparation, particularly in relation to children and their parents’ information-seeking behaviours and preferences when coming to hospital for a planned procedure. Despite increased evidence of the importance of information and preparation for children (Duff et al., 2012; Jaansite et al. 2007) and an increased availability of information, there remain challenges in how children and their parents seek, receive and engage with procedural information. The majority of children and their parents in this study reported that they did not know enough about what would happen when they came to hospital. Although this is concerning, it is not wholly unexpected as previous research shows that children and their parents have poor knowledge levels about many different aspects of hospital care including procedures (Bray et al., 2019a, 2019b; Smith and Callery, 2005), what to expect during an appointment (Hopia et al., 2005), who they can ask if they have questions (Young et al., 2003) and who delivers what care (Callery, 1997).

What this research does add is further understanding of the information preferences and information-seeking behaviours of children and parents before coming to hospital for a procedure. This study aimed to add to the shortage of research concerned with the reasons ‘people do not gather or access health information’ (Kim, 2015, pg120). Both children and their parents in this study reported wanting more information about the planned procedure and so did not display avoidant behaviour, such as wanting limited or no information (Eheman et al., 2009). However, despite a clear need and want for more information, parents and children reported not seeking, accessing or reading the information provided before their hospital visit, creating an ‘information hole’. Although the majority of these children and parents did not display avoidant behaviour, neither did they display active health information seeking (Kim, 2015). The qualitative interviews help shed light on this, by indicating that the children and parents expected key information about procedures to be provided verbally, face to face by health professionals when they arrive at the hospital. This reliance on information from health professionals is reflected in previous research where parents whose child was undergoing surgery also preferred information to be delivered verbally at the hospital (Aranha and Dsouza, 2019) and parents of children with cancer who viewed health professionals as their primary source of information (Kilicarslan-Toruner and Akgun-Citak, 2013).

Children and parent’s expectation for procedural information exchange and preparation on arrival at hospital creates challenges for two reasons. Firstly, family’s expectations may not match the realities of busy health professionals who are working in time-constrained environments and have limited opportunities to spend time explaining procedures and preparing children. Previous investigations into information provision have focussed on instances where there are more opportunities for contact between families and health professionals, for example, surgical procedures (Smith and Callery, 2005), inpatient children (Lambert et al., 2013) and children living with long-term or chronic conditions (Hummelinck and Pollock, 2006; Kilicarslan-Toruner and Akgun-Citak, 2013). In this previous work, if parent’s information needs were not met by health professionals, parents would then seek alternative sources of information (Kilicarslan-Toruner and Akgun-Citak, 2013); this is not an avenue open to children and parents who are attending hospital for a short outpatient procedure. Secondly, there is strong evidence to indicate that children benefit most from preparatory information delivered a few days prior to a procedure or intervention (Jaaniste et al., 2007). The ability of children to understand and use information to meaningfully shape their procedural experience is reduced if information is either not provided or is only delivered immediately prior to a procedure taking place.

It seems that there is further work to be done to promote the benefits of accessing and engaging with information sources, *before coming to hospital,* to children and their parents. As many of these children are not diagnosed with a serious, long-term or chronic condition, they and their parents are less likely to be active information seekers (Kim, 2015) and therefore require health services to signpost them to information resources as part of a multi-method preparation approach. Children and their parents would benefit from pre-procedural resources which can figuratively ‘speak to them’ and deliver information in a way which is engaging and meets the needs of families. Evidence shows that ‘people have a strong preference for information that comes directly from other people’ (Case et al., 2005, pg 358), whether this be face-to-face verbal information, or by engaging with materials which offer a ‘social presence’. Technology as a platform to deliver engaging and interactive preparatory information as an adjunct to face-to-face verbal interaction has potential to improve children and parents’ access to information before a procedure from within their own home. This engaging information should be supplemented by children and parents being afforded the opportunity and time to talk with health professionals and ask questions in order to individualise the procedural information and clarify meanings and processes (Bray et al., 2019a, 2019b) and facilitate children to be active information seekers (Bray et al., 2019a, 2019b; Lambert et al., 2008).

We recognise that while our findings indicate that the vast majority of children do want more information about what will happen during their hospital procedure, there was a small proportion who did *not* want to know. This is congruent with other work which shows that a ‘one size fits all’ approach to providing information to children and parents does not work (Lambert et al., 2008; Steer et al., 2011); this again demonstrates the need for children and their parents to be able to engage with health professionals to discuss their information preferences and needs.

## Limitations to the study

This study had a number of limitations. Firstly, although the sample included children of different ages, genders and ethnicities, this was a small convenience sample, and the study was only conducted within one hospital trust. Secondly, the children and their parent/carers were recruited as the usual care (control) arm of a larger interventional study, and this may have influenced those who chose to participate. Thirdly, the study only used a single-item scale to measure perceptions of procedural knowledge, and the use of multi-item scales may have provided more robust information. Lastly, as the study design was child-centred and focussed on the reports and experiences of children, we did not collect parents’ self-report of their information-seeking behaviours.

## Implications for practice

In order to address the ‘information hole’ reported by children and their parent/carers, health services and professionals need to actively signpost children and their parent/carers to meaningful, accessible and interactive information resources to help prepare children at home prior to planned procedures. Health professionals also need to provide time and actively encourage children and their parents to talk through their understandings of what will happen during their hospital visit and ask questions prior to a procedure being conducted.

## Conclusion

This study has shown that there is currently a mismatch between how children and parents are provided with procedural information and their information-seeking behaviours, expectations and preferences for information. In order for this ‘information hole’ to be filled, there needs to be a concerted effort to develop and systematically use engaging, meaningful and trusted information materials. These information materials need to be directly targeted at children in recognition of their rights and abilities to gain skills in being active information seekers. Children and their parents then need to have the opportunity to clarify and explore their knowledge with health professionals in order to individualise and reinforce their understanding.
